# The role of preserved bowel and mesentery fixation in apple-peel intestinal atresia

**DOI:** 10.1186/s12887-022-03475-z

**Published:** 2022-07-11

**Authors:** Jinbao Han, Zenghui Hao, Long Wang, Ting Yao, Wei Fan, Zheng Zhao, Liuming Huang, Zhilin Xu

**Affiliations:** 1 Department of Pediatric Surgery, The First Affiliated Hospital of Harbin Medical University, No.23 Youzheng Street, Nangang District, Harbin, 150007 China; 2 Department of Neonatal Surgery, Senior Department of Pediatrics, the Seventh Medical Center of PLA General Hospital, No.5 Nanmencang Road Dongcheng District, Beijing, 100700 China

**Keywords:** Jejunal atresia, Apple-peel atresia, Mesoplasty, Retrospective study, Neonatal surgery

## Abstract

**Objective:**

This study explored the feasibility of mesoplasty with end-to-side anastomosis in the treatment of different apple-peel mesenteric defects with high jejunal atresia.

**Methods:**

A retrospective analysis was performed on 42 premature infants admitted to the hospital between 2014 and 2021. Prenatal ultrasound scans revealed bowel dilatation. The patients experienced vomiting after birth and produced white or no meconium. Plain radiography showed double or triple bubble signs and the patients underwent emergency laparotomy. High jejunal atresia with different apple-peel atresia appearance was discovered intraoperatively, involving mobilization of the ileocecal region. Patients received end-to-side anastomosis between the enlarged blind pouch and atretic bowel, as well as mesoplasty. A jejunal feeding tube was placed trans-nasally. Patients were discharged after achieving full enteral feeding. We also reviewed the literature on the subject.

**Results:**

Three patients died and 39 survived. The discharged patients were followed up for 12 months, and none showed post-operative complications such as intestinal obstruction, malnutrition, or chronic diarrhea. All surviving patients reached the expected height and weight for children of the same age.

**Conclusion:**

For cases of high jejunal atresia with apple-peel intestinal atresia, mesoplasty may be a good option to avoid postoperative volvulus.

## Introduction

Jejunoileal atresia (JIA) causes congenital neonatal intestinal obstruction with an incidence of 1.6–3.4/10,000 [1, 2]. Over the past 20 years, improvements in surgery, anesthesia, and preoperative nursing techniques have significantly increased the post-operative survival rate of patients with intestinal atresia [3–6]. Etiological research has identified that JIA is caused by an intrauterine vascular accident, which is also the basis for its distinct pathophysiology from other intestinal atresias [7]. Apple-peel intestinal atresia (APA) is a rare form of small bowel atresia in which the proximal bowel is dilated and the distal bowel wraps around its independent blood supply in a spiral resembling an apple peel, accompanied by mesenteric agenesis. APA accounts for 5% of all intestinal atresias [8].

Generally, the types of common APA are treated by excising the section of the bowel with the vascular malformation and atresia, followed by anastomosis on the normal bowel. When the distal atresia bowel is too short, complete resection of the malformed bowel leads to the occurrence of long-term short bowel syndrome. Careful handling of the distal atretic bowel becomes critical. Total apple-peel atresia (TAPA) and partial apple-peel atresia (PAPA) involve mesenteric dysplasia and vascular malformation. Since the bowel cannot be completely resected through surgery and the management of the mesentery is problematic, the mobilized mesentery can increase the post-operative risk of volvulus and ischemic necrosis. Thus far, only a few studies have reported on APA, and none have introduced the mesopexy method. This is the first time to introduce the technology of mesopexy. In this study, a retrospective analysis was performed on clinical data from 42 patients admitted to the pediatric surgery unit of the First Affiliated Hospital of Harbin Medical University and the Department of Neonatal Surgery, Senior Department of Pediatrics at the Seventh Medical Center of PLA General Hospital. Based on this, we summarized the clinical features of the disease and introduced the mesopexy method for the treatment of APA.

## Methods

### Clinical data

**Fig. 1:**
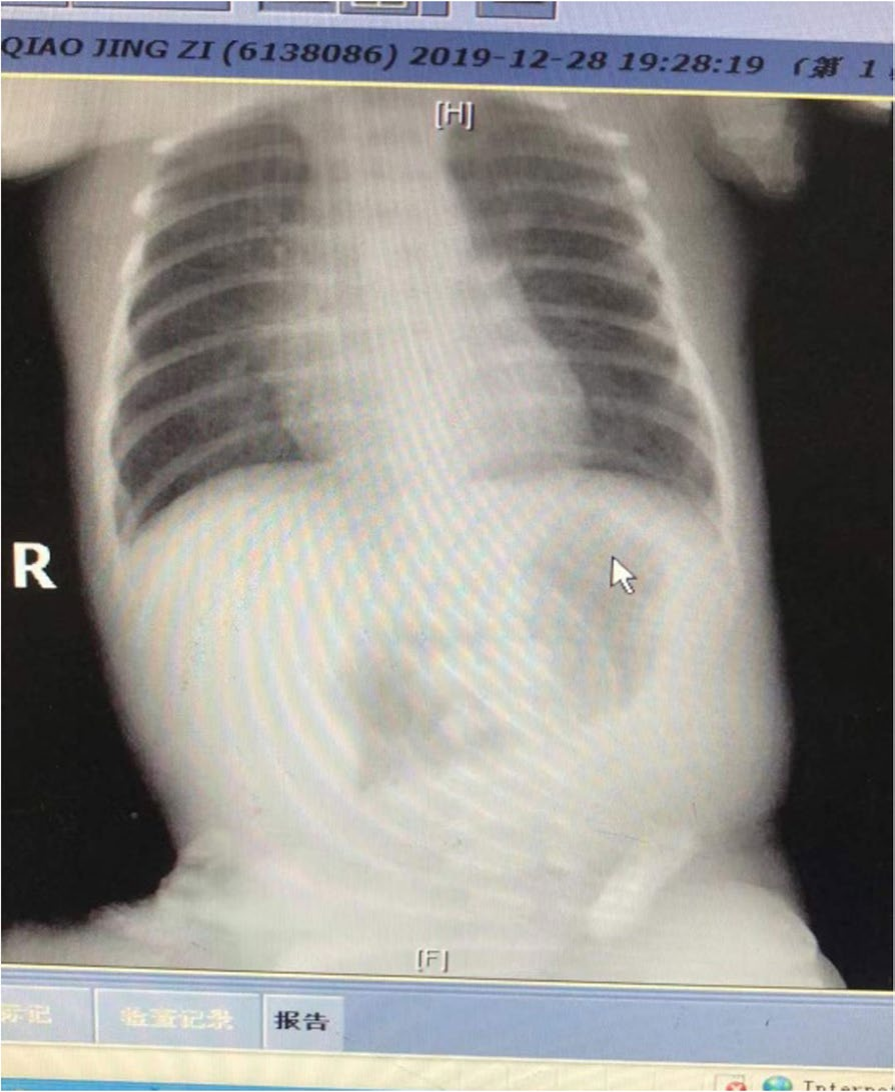
Double bubble sign seen in preoperative X-ray

This study was approved by the Ethics Committee of the First Affiliated Hospital of Harbin Medical University and the Seventh Medical Center of PLA General Hospital. It included 42 cases of premature infants (25 males, 17 females), admitted between 2014 and 2021. As their prenatal ultrasound suggested, with the fetal bowel dilatation and a high volume of amniotic fluid, they were suspected of having digestive tract malformations involving duodenal atresia or high jejunal atresia. The patients presented with vomiting within six hours after birth and dark green gastric contents were removed through gastrointestinal decompression. No meconium or only a small amount of white meconium was expelled. Plain abdominal radiography 24 h after birth showed the double or triple bubble sign (Fig. 1), and postnatal ultrasound scans revealed that the proximal bowel was markedly dilated and the distal bowel was poorly inflated.

The patients underwent emergency laparotomy at the hospital, and high jejunal atresia (about 10 cm in length, located at the start of the jejunum) was found intraoperatively, with significant bowel dilatation and enlargement. APA had occurred at the distal bowel, ranging from the start of the jejunum to the end of the ileum. There were six cases of TAPA, nine cases of partial type, and 27 cases of a common type. With the TAPA cases, the entirety of the small intestines was affected, and the ileocecal region was in a mobilized state. The whole length of the atretic bowel was supplied by only one blood vessel. PAPA refers to the distal atresia of the intestine that needs to retain part of the intestinal malformation—the normal small intestine is very short, and the position of the ileocecal is fixed. The common type of APA refers to the malformation that can be completely removed, and the remaining small bowel length is sufficient to support growth and development. The surviving patients were followed up for 12 months after discharge, and none showed post-operative complications such as intestinal obstruction, malnutrition, or chronic diarrhea. All surviving patients reached the expected height and weight for children of the same age.

### Surgical procedure

**Fig. 2:**
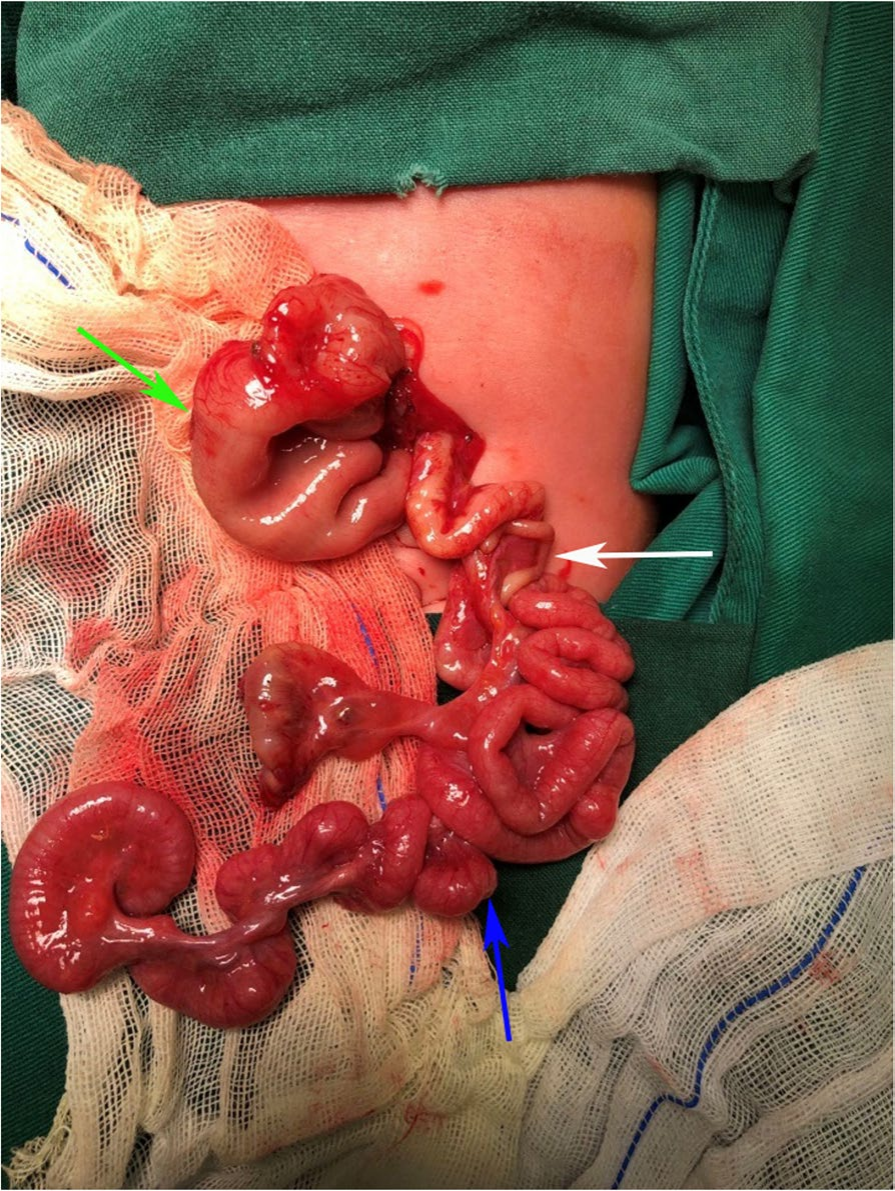
Green arrow: enlarged blind pouch (jejunum); white arrow: appendix; blue arrow: apple-peel atresic bowel

**Fig. 3:**
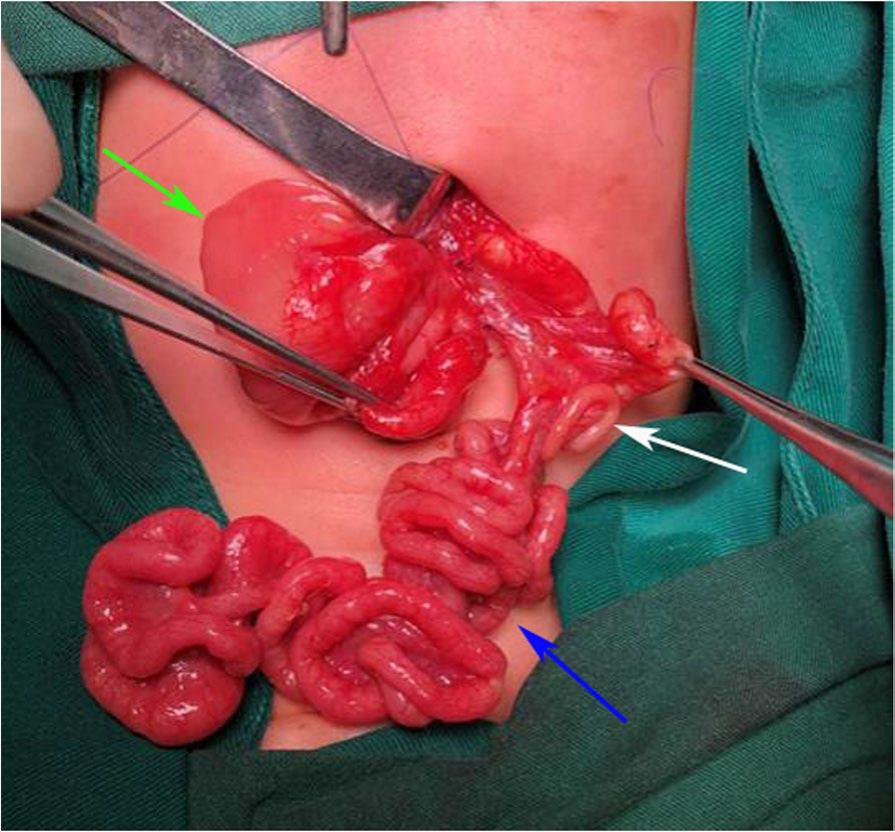
Green arrow: jejunum; white arrow: appendix; blue arrow: small intestines

**Fig. 4:**
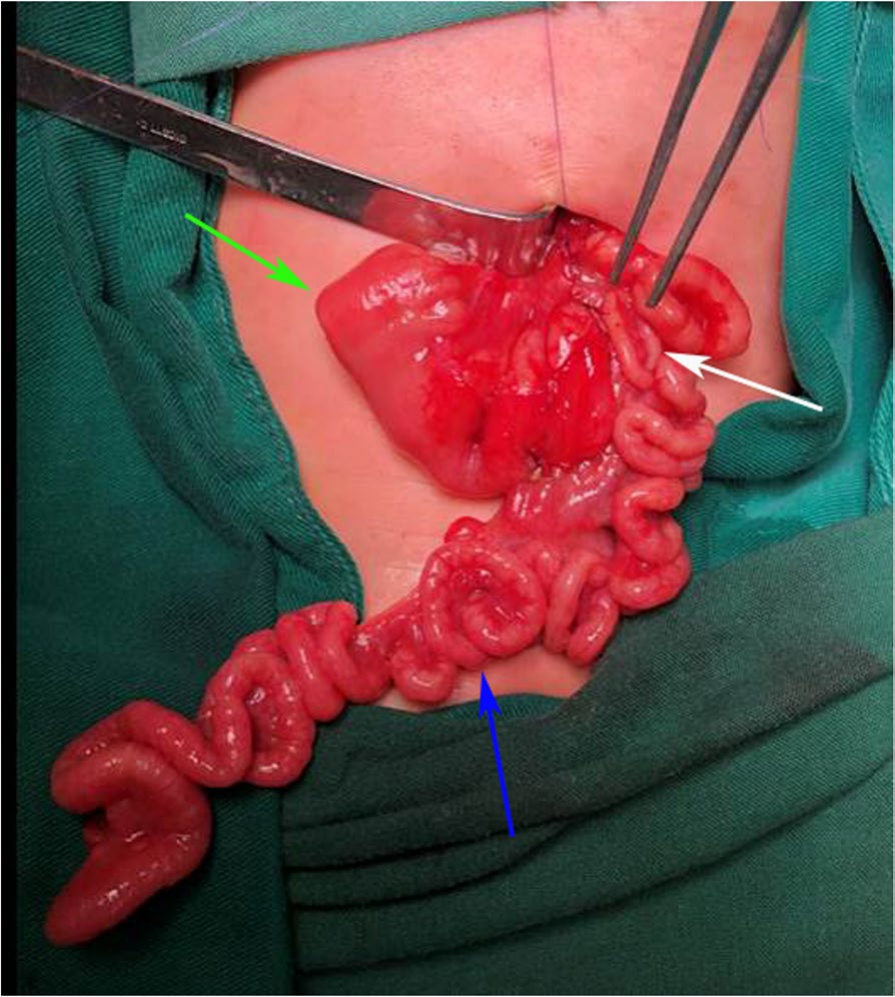
Green arrow: jejunum; white arrow: appendix; blue arrow: small intestines

**Fig. 5:**
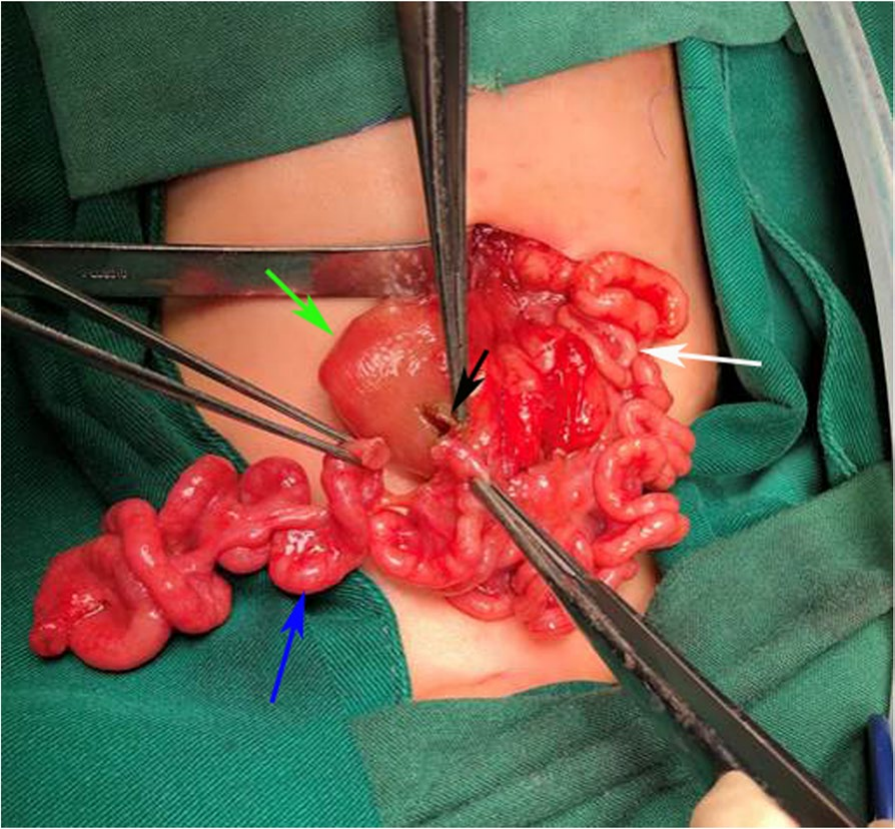
Green arrow: jejunum; white arrow: appendix; blue arrow: resected small intestines; black arrow: anastomosis

A transverse incision was made in the upper abdomen, and exploration of the abdominal cavity revealed a proximal high jejunal atresia, with dilatation of about 10 cm in length. The length of the normal small intestine in the common type of APA is sufficient to allow complete resection of the malformed bowel. The proximal enlarged bowel was longitudinally dissected and anastomosis was performed, which will not be described in detail. TAPA was found in the distal bowel, and the appendix was located in the left upper abdomen. The whole apple-peel atresia bowel was supplied by only one blood vessel (Fig. 2), and we made sure to protect the only blood supply from the superior mesenteric artery. Warm saline was injected into the blind pouch of the atresia, and the patency of the distal bowel was examined. Normal saline through the anus indicated good patency of the distal bowel and the absence of other malformations. The enlarged bowel at the blind pouch was fully preserved. Retrograde fixation of the small bowel mesentery was then performed, starting from the ileocecal region and proceeding around the enlarged blind pouch (Fig. 3). After fixation of the ileocecal region, the malformed small bowel mesentery was fixed in a proximal direction along with the enlarged bowel. During the procedure, care was taken to ensure that the blood vessel remained tension-free to minimize traction on the blood vessel by the bowel, and an adequate length of the small intestine was preserved (Fig. 4). Once the proximal end of the enlarged bowel was fixed, the excess atresia bowel was resected, and the enlarged bowel was incised at the corresponding position. The incision was of the same diameter as the oblique incision at the distal atretic bowel. This was followed by end-to-side anastomosis (Fig. 5).

**Fig. 6:**
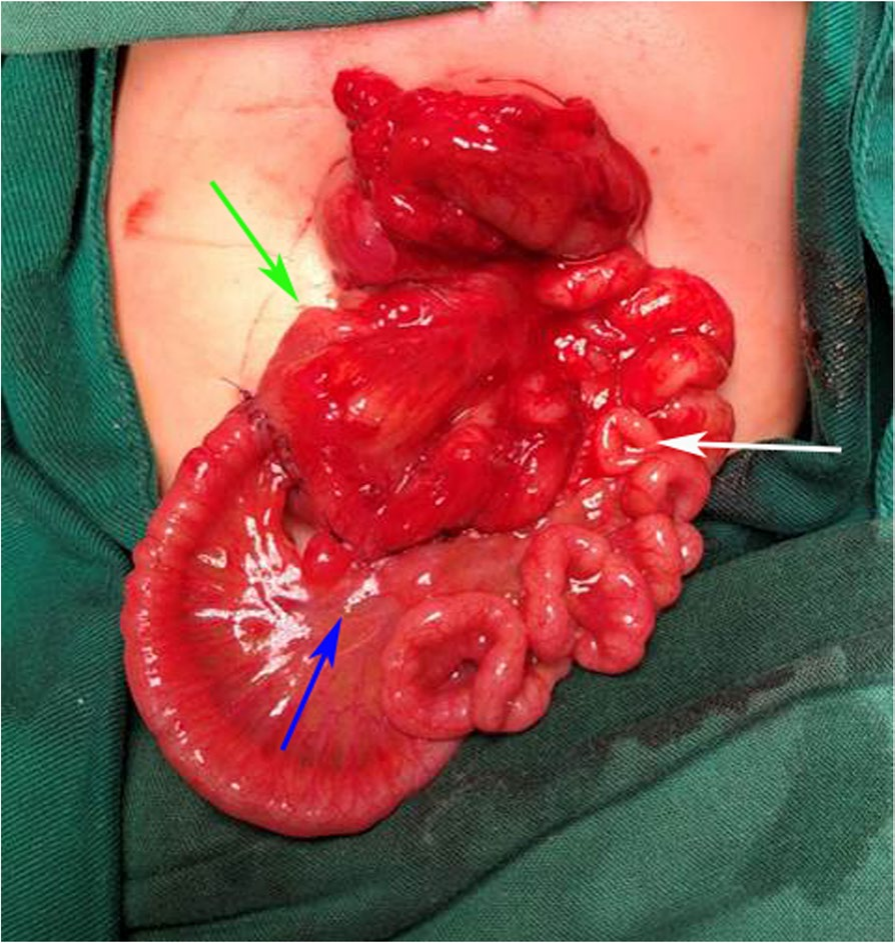
Green arrow: jejunum; white arrow: appendix; blue arrow: shaped mesentery

A jejunal feeding tube was placed trans-nasally, and an additional 10 cm was inserted after passing through the anastomosis. This enabled the provision of early enteral nutrition and anastomotic support (Fig. 6). The key points of mesoplasty were as follows: The apple-peel atresia bowel was returned to its original position, and the superficial fascia of the feeding vessel was sutured with 5–0 absorbable thread according to the length of the atretic bowel. We made effort to protect the superior mesenteric artery. The enlarged and dilated bowel served as the centre for the fixation of the atretic bowel mesentery, which became the new mesentery of the enlarged bowel, thus preventing intestinal volvulus, angulations, and other complications.

## Results

### Socio‑demographic characteristics of participants

All patients underwent end-to-side anastomosis and mesoplasty for jejunal atresia with TAPA and PAPA. The common type underwent bowel resection and anastomosis. Three patients died of severe infection and kidney failure (TAPA 1 (16.7%), PAPA 1 (11.1%), common type of APA 1 (3.7%), while 39 survived. The average gestational age of the three types were, in weeks, 33(31–35), 33(32–35) and 33(31–35), respectively, and the average birth weights in grams were 2001(1570–2410), 1864(1580–2270) and 2418(1580–2970). The average operative times were 89(65–120) minutes, 92(60–120) minutes and 87(60–120) minutes. The post-operative duration of parenteral nutrition ranged from 59(3–128) days to 70(6–132) and 51(15–110) days, while the duration of enteral nutrition varied from 47(0–131) to 67(0–118) and 46(5–101) days, respectively. The duration of hospital stays ranged from 71(3–132) to 92(3–143) and 74(15–129) days for the respective types. The discharged patients were followed up for 6–12 months, and none showed post-operative complications such as intestinal obstruction, malnutrition, or chronic diarrhea. All surviving patients reached the expected height and weight for children of the same age. See Table 1 for the details.

**Table 1:** Clinical data of pediatric patients

*n* = 42	Partial apple-peel atresia^a^ (*n* = 9)	Total apple-peel atresia^b^ (*n* = 6)	Common apple-peel atresia^c^ (*n* = 27)
Gestational age (weeks)	33(31–35)	33(32–35)	33(31–35)
Birth weight (grams)	2001(1570–2410)	1864(1580–2270)	2418(1580–2970)
Operative time (minutes)	89(65–120)	92(60–120)	87(60–120)
Enteral nutrition time (days)	47(0–131)	67(0–118)	46(5–101)
Parenteral nutrition time (days)	59(3–128)	70(6–132)	51(15–110)
Length of hospital stay (days)	71(3–132)	92(3–143)	74(15–129)
Outcome (cured n, n%)	8(88.9%)	5(83.3%)	26(96.3%)

^a^Partial apple-peel atresia: This type of apple-peel atresia refers to the malformed bowel that cannot be completely removed. The length of the normal small intestine is less than 60 cm, and most of the malformed intestinal bowel needs to be preserved

^b^Total apple-peel atresia: This type of apple-peel atresia refers to the malformed bowel, and all of the intestines and the ileocecal region are in a mobilized state. There is no normal small bowel. The whole length of the atresic bowel was supplied by only one blood vessel, and the whole malformed intestinal could not be resectioned

^c^Common apple-peel atresia: This type of apple-peel atresia refers to a malformed bowel that can be completely resected, with the remaining normal small bowel length being longer than 60 cm

We searched the literature for nearly 10 years and found 10 reports on the total apple-peel atresia [10–19]. The summary found 3 deaths, the mortality rate of 30%, and 3 cases of short bowel syndrome resulting in growth difficulties. 1 case developed intestinal obstruction after operation, while 2 cases were only followed up until discharge (Table 2).

**Table 2:** Outcomes of reported cases

Author	year	Sex	GA	BW	Procedure	Complication	outcome	Follow up
Weber et al.[10]	1999	F	36	2100	End-to-end anastomosis	SBS, Intestinal failure	Survived	Mild failure to thrive
Arbell et al. [11]	2006	M	32	1500	End-to-end anastomosis	Intestinal obstruction	Survived	Six months, the baby was well and thriving
Tatekawa et al. [12]	2007	F	36	2104	Side-to-side anastomosis	SBS, Intestinal failure	Survived	6 months old and 6230 g,under the care of a local hospital
Ahmed et al. [13]	2009	M	34	1200	End-to-end anastomosis	Sepsis,	Died	-
Patil et al.[14]	2011	F	33	1600	Duodenojejunostomy,Release of Ladd’s bands &inversion Appendectomy	Sepsis,	Died	-
Altokhais et al.[15]	2014	M	33	1900	Side-to-oblique anastomosis & Release of Ladd’s bands	-	Survived	16 months, no gastrointestinal issues
Alnosair et al.[16]	2014	F	31	1400	End to end anastomosis	-	Survived	Follow to Discharge
Pathak et al.[17]	2014	-	33	1300	End to oblique anastomosis	-	Survived	Follow to Discharge
Saša et al.[18]	2016	M	29	1240	End-to-end anastomosis	Cardiorespiratory failure	Died	-
Kirtane et al.[19]	2019	M	36	2250	End-to-end anastomosis	SBS	Survived	2 years old and weighs only 11 kg

## Discussion

APA is a very rare disorder and an extremely serious condition in neonates. It is more common in premature infants and is often accompanied by short bowel, multiple atresias, apple-peel configuration and other related malformations, resulting in numerous difficulties during treatment. Furthermore, it has a much higher incidence of post-operative complications and mortality rate than other types of small bowel atresia [9]. The incidence of high jejunal atresia with APA, as reported in this study, is even rarer, and search though the existing English literature revealed only 10 case reports of apple-peel atresia thus far [10–19], with a high mortality rate.

The current accepted treatment option of the APA is resection of the malformed intestinal, but, when apple-peel removal poses a risk of short bowel, part of the apple-peel intestinal tube has to be preserved. However, there is a risk of post-operative volvulus after preservation. At present, there is no literature report on the preservation of the apple-peel atresia. In fact, surgeons are concerned that simply preserving the malformed bowel can potentially lead to postoperative volvulus or ischemic changes. There is no report on how the risk of post-operative volvulus can be avoided. Therefore, it is safer and more reliable to preserve the diseased bowel and fixation of the mesentery which is no literature report on how to preserve the diseased bowel to prevent volvulus. The surgical method reported in the literature is end-to-end/-oblique or side-to-side/-oblique anastomosis, and the specific surgical method is not given in detail. Post-operative complications include SBS or bowel failure, intestinal obstruction, which may be caused by excessive bowel resection and lack of good fixation of the mesentery. Three of the surviving cases had feeding difficulties and failure to thrive, which are associated with short bowel syndrome or intestinal failure. We analyzed the reason that the remaining intestinal is too short, or the development of intestinal function is not enough. Lack of good mesoplasty fixation after complete resection of APA may lead to post-operative intestinal obstruction and prolong treatment time. The time of follow-up reported in the literature is variable, and long-term follow-up results are lacking. The cure rate after mesenteric fixation in this group was 86.7%. These groups of patients were followed up for 1 year after the growth and development were good. We consequently summarized the APA case and found that mesenteric fixation is a feasible surgical approach.

There are currently no publications on the surgical treatment of apple-peel atresia for TAPA in particular. Patil et al. [20] reported on the maturity and importance of end-to-end linear anastomosis in treating different types of intestinal atresia, demonstrating that this technique significantly reduced mortality and post-operative complications. For common APA, the remaining length of the normal intestinal tube is sufficient, and the intestinal malformed bowel can be completely removed without short bowel syndrome after surgery. The remaining intestinal length is less than 60 cm, and it is particularly important to preserve the apple-peel atresia. Preservation of malformed apple-peel atresia length is important, especially in TAPA and PAPA. Fixing the mesentery stabilizes the position of the Intestinal tube and reduces excessive and irregular movement of the intestinal tube after food stimulation. We have summarized the surgical techniques employed in 42 cases of this rare disorder that were admitted to our hospitals over seven years in the present study. The key points to note in pursuit of good post-operative recovery are:

1. The proximal atresia bowel began at the jejunum, the position of the atresia was relatively high, and the position of the proximal jejunum was relatively fixed.
2. Mesopexy was performed around the atresia blind pouch.
3. Mesoplasty was not performed on the blind pouch, which posed a high risk to the patency of the end-to-side anastomosis to enlarge the blind pouch. The placement of a nasojejunal feeding tube could therefore reduce the risk of anastomotic obstruction.

First, after mesoplasty was performed in this group of patients, the mobility of the enlarged blind pouch was relatively low due to the high position of the proximal atresia. Hence, the volvulus potential not occurs once the mesentery has been fixed. Fixing the position of the proximal bowel can reduce the traction on the only supplying blood vessel, while associated with preventing the volvulus, incarceration, and necrosis of the distal bowel. Second, due to the high mobility of the distal bowel and the absence of mesentery to fix the bowel loop, the proximal enlarged blind pouch became the preferred choice for fixing the mobilized distal bowel. This can also reduce the traction on the superior mesenteric artery and associated with reduce volvulus, without affect the blood supply of the distal bowel. The risk of volvulus and necrosis in the distal bowel might be also reduced after feeding. Finally, the placement of a nasojejunal feeding tube enabled the early use of the distal bowel and accelerated the recovery of intestinal function, while also providing good support for the anastomosis and reducing the risk of anastomotic stenosis. Since a patient with APA has an adequate length of distal bowel, partial resection of the affected bowel will not affect the overall bowel length and will not lead to the occurrence of short bowel syndrome.

The advantages of mesopexy were significantly highlighted in the case of total APA. Fixing the only blood vessel supplying the small intestines around the proximal atresia, enlarged and dilated bowel, and reshaping the mesentery not only reduced the irritation on the only blood vessel, but also reduced the mobility of the small intestines. Fixation was performed from the ileocecal region along with the distal enlarged bowel, while fixing the apple-peel atresia small intestines around the lateral wall of the enlarged bowel, which can reduce the formation of bowel incarceration, volvulus, and internal hernia caused by rapid peristalsis. This technique improved the prevention of post-operative complications.

This group of patients exhibited total apple-peel atresia of the distal bowel and had an adequate length of distal small bowel. However, owing to the large disparity in the diameters of the proximal and distal bowels and the presence of an enlarged blind pouch, patients are more prone to food retention and prolonged post-operative enteral and parenteral nutrition. The issue of short-term retention can therefore be solved by the placement of a nasojejunal feeding tube. As the distal bowel gradually developed and widened, the issue of food retention was resolved, and good recovery was also observed in intestinal function during follow-up examinations. For the malformation bowel that can be completely resected in patients with the common type of APA, the tailoring and shaping of the anastomosis are also crucial. The mesentery of the remaining bowel can be developed, but it still needs good fixation and arrangement. When the PAPA malformed bowel needs to be partially preserved, a good fixation of the mesentery can reduce post-operative volvulus. There is a sufficient length of the intestinal tube left to absorb nutrients and avoid the occurrence of short bowel syndrome. Three patients died of severe infection and kidney failure after the surgery. The remaining 39 patients had good post-operative prognoses. The surgical technique may well have contributed to good surgical outcome, but there could be multifactorial contributions even in this current cohort.

There are some limitations to this study. First, a retrospective analysis was conducted in this study, which may have led to limitations in data analysis. Second, our total sample size was relatively low, and more cases are needed for analysis. Third, the surgical method of surgery still needs a large number of cases to prove the feasibility, and the indicators that affect the success of the surgery are also the result of multi-factor contributions. Postoperative nutritional support also needs to be verified by a large sample size and multi-center data.

## Conclusion

Intraoperative mesopexy is a safe and effective method for the treatment of TAPA and PAPA, and it may be potential benefit improvement of possible postoperative volvulus.

## Data Availability

The datasets used and/or analyzed during the current study are available from the corresponding author on reasonable request.

## Abbreviations

JIA: Jejunoileal atresia
APA: Apple-peel intestinal atresia
TAPA: Total apple-peel atresia
PAPA: Prtial apple-peel atresia
